# “We were the best people to do the job”: Caregivers’ reported outcomes of a virtual caregiver-delivered program for autistic preschoolers

**DOI:** 10.1177/23969415241244767

**Published:** 2024-04-30

**Authors:** Lauren Denusik, Danielle Glista, Michelle Servais, Jodi Friesen, Janis Oram, Barbara Jane Cunningham

**Affiliations:** 6221 School of Communication Sciences and Disorders, University of Western Ontario, London, ON, Canada; University of Western Ontario, London, ON, Canada; 6221 School of Communication Sciences and Disorders, University of Western Ontario, London, ON, Canada; Thames Valley Children's Centre, London, ON, Canada; 6221 School of Communication Sciences and Disorders, University of Western Ontario, London, ON, Canada; School of Communication Sciences and Disorders, University of Western Ontario, London, ON, Canada; School of Communication Sciences and Disorders, University of Western Ontario, London, ON, Canada; CanChild Centre for Childhood Disability Research, Hamilton, ON, Canada

**Keywords:** Autism, caregiver-delivered programs, virtual services

## Abstract

**Background and aims:**

Caregiver-delivered programs are a recommended best practice to support young autistic children. While research has extensively explored children's outcomes quantitatively, minimal qualitative research has been conducted to understand caregivers’ perspectives of program outcomes for themselves and their children. Hearing directly from caregivers is an important step in ensuring these programs are meeting the needs of those who use them. This study explored caregivers’ perceived outcomes following one virtual caregiver-delivered program, The Hanen Centre's *More Than Words^®^* (MTW) program*.*

**Methods:**

This study was a secondary analysis of data from individual interviews conducted with 21 caregivers who had recently participated in a virtual MTW program. A hybrid codebook thematic analysis approach was taken to analyze the interview data. Program outcomes were coded and analyzed within the International Classification Functioning, Disability, and Health (ICF) framework. Additionally, caregivers completed an online survey and rated Likert Scale items about perceived program outcomes, which were analyzed descriptively.

**Results:**

Five themes were identified: (1) caregivers learned new strategies to facilitate their child's development, (2) caregivers developed a new mindset, (3) children gained functional communication skills, (4) caregiver–child relationships improved, and (5) caregivers gained a social and professional support network. These themes fell within four of five ICF framework components (activities, participation, personal factors, and environmental factors). No themes were identified under Body Structures and Functions. Survey results indicated most caregivers reported learning new communication strategies (*n *= 20, 95%), and identifying new teaching opportunities with their child (*n *= 21, 100%).

**Conclusions:**

Some reported outcomes, related to Activities and Participation, were consistent with previous reports in the literature on the MTW program. In line with previous research, caregivers learned strategies to support their child's communication development. Contrary to previous quantitative studies, caregivers in this study rarely commented on gains in vocabulary and instead focused on gains in skills that positively impacted their child's ability to engage in meaningful social interaction. Novel outcomes were identified within the Participation, Personal Factors, and Environmental Factors components of the ICF framework.

**Implications:**

Caregivers in this study identified important outcomes for themselves and their child that have not been the focus of prior research, suggesting it is important to integrate their perspectives in the development and evaluation of caregiver-delivered programs. Clinicians should include goals that address outcomes identified as important by caregivers, including those that address children's Participation, and those that target caregivers’ Personal and Environmental Factors. Developers of caregiver-delivered programs could integrate identified goals to ensure they are meeting families’ needs.

Caregiver-delivered programs have been recommended as a best practice to support communication development in autistic preschoolers (Zwaigenbaum et al., 2015). In these programs, a clinician teaches caregivers strategies to facilitate their child's communication development, but the caregiver is the primary person using the strategies with their child in everyday activities (Bearss et al., 2015). In addition to empowering families to support their children's development at home, caregiver-delivered programs can be facilitated in a group setting to help reduce waitlists by increasing the number of families accessing speech and language services (McGill et al., 2020). Caregiver-delivered programs aim to target skill development in young autistic children in a variety of areas, including language, social communication, and play. The outcomes of these programs have been extensively researched, primarily using quantitative research methods (Nevill et al., 2018; Oono et al., 2013), but there is a need to also explore outcomes associated with caregiver-delivered programs qualitatively to better understand participants’ experiences. Work that directly integrates families’ perspectives would help ensure that these programs meet the needs of their end users. It could also help guide research by ensuring scientists consider and measure outcomes that are most important to families, which is key to the development of family-centered care.

## Family-centered care

Families are critical members of the team supporting an autistic child. Family-centered care highlights the important role of caregivers in providing healthcare services, in particular that caregivers should have opportunities to decide on the goals and programs they wish to access to support their child (Rosenbaum et al., 1998). Family-centered care assumes that caregivers spend the most time with their child and thus are most familiar with their child's needs (Rosenbaum et al., 1998). Other key components of this approach to care include respectful communication that focuses on the strengths of the child and the ongoing collaboration between caregivers and healthcare professionals (Kokorelias et al., 2019). An important benefit is caregivers feeling empowered to support their child (Fordham et al., 2012). While many providers aim to employ family-centered care, caregivers have expressed that they do not always feel their needs are taken into account and, as a result, are receiving services that do not fit the individual needs of their families (Pozniak et al., 2023). Specific to caregivers of autistic children, it has been found that caregivers value healthcare professionals who are responsive to the individual needs of their child, recognizing the diverse presentation of autism and that the same approach cannot always be utilized (Kouo et al., 2022). While caregiver-delivered programs may encompass the many values of family-centered care, such as having the caregiver actively involved in determining goals and implementing services, another important component is taking time to listen to their experiences. In an effort to provide family-centered care it is critical that researchers and clinicians understand the outcomes of the program, and if they meet the needs and expectations of families.

## Quantitative outcomes of caregiver-delivered programs

Many existing caregiver-delivered programs for autistic preschoolers have been evaluated quantitatively through randomized controlled trials and systematic reviews. In most cases, the impact of an intervention is assessed using tools that are selected by researchers and used to obtain scores that can be used to make conclusions about effectiveness. For children, reported outcomes include improvements in language and social communication skills (Althoff et al., 2019; Cheng et al., 2023; Oono et al., 2013). Specifically in the social communication category, positive outcomes include gains in joint attention, child initiation and engagement, and improved caregiver–child interactions (Althoff et al., 2019; Oono et al., 2013). The impact of these programs on language is less clear. While one systematic review reported no difference in children's postintervention language skills (Oono et al., 2013), findings from other suggested programs may significantly impact children's language (Cheng et al., 2023). Possible explanations for this discrepancy include differences in the specific caregiver-delivered programs assessed and differences in the assessment tools used. Research has also used quantitative tools to explore outcomes for caregivers who participate in caregiver-delivered programs. Reported benefits for caregivers include an improved ability to implement language facilitation strategies, improved interactions with their children, and reduced stress (Lichtlé et al., 2020; Noyan Erbaş et al., 2020; Oono et al., 2013; Shalev et al., 2020).

Tools such as the Early Social Communication Scales (Mundy et al., 2003) and Vineland Adaptive Behavior Scales (Sparrow et al., 2005) are often used to quantitatively assess the impact of caregiver-delivered programs on children's communication skills. A challenge in the field of autism intervention research is that outcomes are not measured using consistent tools (McConachie et al., 2015). For example, in their systematic review, McConachie et al. (2015) reported 131 different outcome tools used in 184 autism intervention studies, and these were used to assess outcomes that fell into twelve different measurement categories, (e.g., language, attention, social communication). The wide variety of tools used to measure quantitative outcomes may, in part, be related to a lack of agreement among researchers regarding the important outcomes of autism interventions (McConachie et al., 2018), as well as the complexity and variability in presentation between autistic children. The variability in tool use also presents challenges in synthesizing evidence to assess treatment efficacy. Additionally, there is concern that the quantitative measures used in autism research are not specifically designed to assess autism characteristics and, therefore, may not be sensitive enough to accurately capture the specific changes made by autistic children in these programs (Grzadzinski et al., 2020). The reported challenges with quantitative measures present an issue for researchers and clinicians wanting to understand the impact of specific interventions and whether an intervention is likely to meet the unique needs of young autistic children and their families.

Although there has been extensive quantitative research on caregiver-delivered programs, the everyday impact remains unclear, including the strategies caregivers find most beneficial and the impact on children's social interactions with peers and family members. Such outcomes are not as frequently reported in the literature because they can be hard to measure; however, caregivers of autistic children have reported them as being highly important (McConachie et al., 2018; Pituch et al., 2011). Qualitative research methods may be helpful in addressing some of these critical knowledge gaps.

## Qualitative outcomes of caregiver-delivered programs

To further understand the impact and improve the effectiveness of caregiver-delivered programs for autistic preschoolers, it is critical that researchers and clinicians seek out and report on families’ observed outcomes and perspectives (Swigert & Wright, 2020). Identifying what matters to caregivers is important in providing family-centered care (Hirpa et al., 2020) and exploring their experiences using qualitative approaches is an important step in understanding valued outcomes so programs can be developed to meet their needs (Jurek et al., 2023). A recent review by Jurek et al. explored the experiences of families in caregiver-delivered programs by synthesizing the existing qualitative research on facilitators and barriers to caregiver participation. One of their four identified themes described the positive outcomes of caregiver-delivered programs, including caregivers learning new skills and feeling empowered to support their child. As part of this theme, researchers briefly touched on outcomes for the child, including gains in verbal and nonverbal communication; further exploration was noted as a need when related to outcomes beyond social communication skills, in addition to seeking the caregivers’ perspectives. While this review synthesized the existing research on facilitators and barriers, the authors only briefly touched on caregiver-reported outcomes. This lack of detail may be because it was out of scope for the review, or it may be due to the limited body of research on caregiver-reported outcomes. It is critical to dig deeper into caregiver-reported outcomes to further understand what families gain from participating in caregiver-delivered programs, particularly because there may be important perceived outcomes that have not been studied.

## The More Than Words program

Many published caregiver-delivered programs are available to families of autistic children, and any could be used to begin to explore caregivers’ perceived outcomes. *More Than Words^®^ (MTW)—The Hanen Program for Parents of Autistic Children or Children Who May Benefit from Social Communication Support* (Sussman et al., 2016) was selected for this study because (a) it was commonly accessed by caregivers in Ontario, Canada, where this study was conducted (Binns et al., 2022); (b) our team is highly familiar with the program; and (c) we had previously collected caregiver interviews related to the newly adapted virtual version of the program (Denusik et al., 2023).

MTW is led by a speech-language pathologist (SLP) who has undergone multiday certification training provided by The Hanen Centre. The program is run by SLPs worldwide (Lok et al., 2021; Sokmum et al., 2017), and locally in Ontario, Canada (Binns et al., 2022). The 13-week program includes a preprogram consultation, eight group sessions, and three individualized video feedback sessions. All sessions are focused on teaching caregivers strategies to support children's social communication and play development. During the two-and-a-half-hour group training sessions, the SLP delivers the program content and helps caregivers develop plans to implement strategies to facilitate communication with their children at home. In the individual video feedback sessions, the SLP observes and records caregivers’ use of strategies during preplanned caregiver–child interactions. Following the recording, the SLP and caregiver review the video, reflect on how the child responded to the caregivers’ use of strategies, and identify areas in which the caregiver could improve strategy use. In addition to the in-person format, a newly developed virtual version of the MTW program is available, which offers families the opportunity to participate in the same program components virtually using video conferencing software (Erdmann et al., 2019).

Quantitative research on the in-person MTW program includes a randomized controlled trial (Carter et al., 2011), case (e.g., Girolametto et al., 2007), and cohort (e.g., McConachie et al., 2005) studies. Children's skills have been assessed using various measures and reported outcomes include gains in social communication, vocabulary, and engagement (Girolametto et al., 2007; McConachie et al., 2005; Prelock et al., 2011; Sokmum et al., 2017). Changes in caregivers’ skills have also been assessed, and caregivers have been shown to increase their responsiveness and use of supportive communication strategies during the program (Girolametto et al., 2007; McConachie et al., 2005; Sokmum et al., 2017). Quantitative outcomes associated with the virtual MTW program have also been explored and were found to be similar to those reported for the in-person program (Garnett et al., 2022a). Notably, the available literature has focused on outcomes associated with children's communication skills and caregivers’ language facilitation skills. Research has not yet explored functional outcomes associated with the MTW program, or outcomes associated with the less-commonly studied components of the International Classification of Functioning, Disability and Health (ICF) framework (e.g., Environmental Factors, Personal Factors). This may be in part due to a lack of available outcome measures to support these types of investigations.

One study has explored outcomes associated with the virtual MTW program. Garnett et al. (2022b) explored caregivers’ perceptions of the virtual program by analyzing their responses on evaluation forms and surveys. This team identified perceived intervention outcomes for caregivers and children, such as caregivers having more confidence to support their child's development, and improvements in children's communication skills. Although this study provided preliminary evidence for caregivers’ perceived program outcomes, it included a small sample size (11 participants), and used surveys with only a few open-ended questions, which may have limited the researchers’ ability to capture all perceived outcomes. To date, no study has explored caregivers’ self-identified outcomes following the MTW program in detail using interviewing. If caregiver-delivered programs aim to follow a family-centered approach, it is critical to understand the outcomes caregivers value so that services can be tailored to meet their needs.

## Current study

This study is a secondary analysis of interview data from work conducted to understand caregivers’ views of the barriers and facilitators to participating in the virtual MTW program during the COVID-19 pandemic (Denusik et al., 2023). While caregivers in Denusik et al. (2023) reported learning new strategies for supporting children's communication as a facilitator to participating in the virtual MTW program, other perceived outcomes were not described. The current study, therefore, aimed to answer the question: What are caregivers’ perceived outcomes for their children and themselves following their participation in the virtual MTW program?

## Methods

### Participants

Twenty-one caregivers from across Canada who completed the virtual MTW program participated in virtual interviews between February and August 2021. The interviews were open to any families in Canada who had recently completed the MTW program. Hanen-trained speech-language pathologists who agreed to facilitate recruitment, shared a flyer at the start of their virtual MTW Program. Interested families then completed an online survey to share their contact information with the research team (see Denusik et al., 2023 for more details). As participation was voluntary, we collected data only for the families who enrolled in the study, and thus cannot report the proportion of families who completed the MTW program overall. Similarly, we cannot report the number of caregivers who enrolled in but did not complete an MTW program. For most families, the participating caregiver was the mother (*n *= 18); however, a father (*n *= 1), aunt (*n *= 1), and grandparent (*n *= 1) were also interviewed. Of the 21 families, 14 had a child with an autism diagnosis, while the rest had social communication concerns. Children ranged in age from 18 to 54 months (*M *= 32.91 months, *SD *= 11.62 months). It should be noted that these families had participated in different MTW groups, delivered by different SLPs. See Table 1 for participant demographics.

**Table 1.** Characteristics of caregivers and their child.

Characteristics	Number (%) of participants
Gender identity of adult participants	
Female	20 (95)
Male	1 (5)
Sex of child participants	
Female	4 (19)
Male	17 (81)
Participants’ ethnic or cultural background (could select more than one)	
Arab/West Asian	1 (5)
Black	1 (5)
White	15 (71)
First Nations	1 (5)
Metis	1 (5)
South-East Asian	1 (5)
Other	4 (19)
Total family income	
Less than $20,000	2 (10)
$40,000 to $59,000	3 (14)
$60,000 to $79,999	7 (33)
$80,000 to $99,999	1 (5)
More than $100,000	8 (38)
Community size	
Small population center (population between 1,000 and 29,999)	5 (24)
Medium population center (population from 30,000 to 99,999)	3 (14)
Large urban population center (population over 100,000)	13 (62)

### Survey data

Prior to the interviews, families were asked to complete an anonymous demographic survey that was developed and administered using Research Electronic Data Capture (REDCap; Harris et al., 2009), a secure data collection system housed at the University of Western Ontario. In addition to demographic questions, participants were asked to rate 16 items about their experience in the virtual program (e.g., to what extent did you get the information you wanted). A 7-point Likert Scale (ranging from “*not at all*” to “*to an extremely great extent*”) was used to capture the range of participants’ experiences. Although there were 16 items, this secondary analysis only reports on the 12 items that focused on caregivers’ perceived program outcomes. The four items not included were related to technology challenges. The 12 included items were analyzed descriptively using frequency counts and descriptive statistics.

### Interviews

We used a semistructured interview approach and developed an interview guide that included 19 questions about caregivers’ experiences participating in the MTW program virtually (see Appendix A for a copy of the interview questions). Open-ended interview questions were developed collaboratively by the research team, which included members with expertise in autism, SLP service delivery, virtual services, and qualitative methodology. All interviews were conducted by the first author, who was not affiliated with the MTW program in any way. The interviews were conducted virtually, using Zoom videoconferencing software (Zoom Video Communications Inc, 2023). An audio recording of the interviews captured through Zoom was transcribed verbatim by a research assistant.

#### Theoretical framework

The World Health Organization's ICF (World Health Organization, 2001) guided the analysis process, which aimed to categorize and describe caregivers’ reported outcomes. We selected the ICF framework and its associated codes for this study to allow for a focus on children's functional outcomes following the MTW program, and because of its ability to capture outcomes related to participation, a significant challenge faced by many young autistic children that is not always formally assessed (Askari et al., 2015). For this study, the ICF framework was also useful for capturing caregivers’ outcomes (e.g., learning to use a new communication strategy = Activities), as well as outcomes associated with caregivers’ Personal Factors (e.g., confidence) and Environmental Factors (e.g., supports) that have not previously been reported in the literature on the MTW program.

The ICF framework includes two parts: (a) Functioning and Disability, and (b) Contextual Factors. Functioning and Disability has three components: (a) *Body functions and structures*: physiological functions and anatomical parts of the body (e.g., production of speech sounds); (b) *Activity*: completing a task or action (e.g., following two-step directions); (c) *Participation*: involvement in various life situations (e.g., communicating with others). There are two Contextual Factors: (a) *Personal Factors*: an individual trait that may impact a person's disability (e.g., age, behavioral disposition, attention); (b) *Environmental Factors*: factors external to the individual that can influence health, well-being, and experiences (e.g., physical, social, and attitudinal environment; Kwok et al., 2022). Each element includes a set of codes that can be used to systematically organize reported functional outcomes in more detail (WHO, 2001) and have been used for qualitative analysis in past research (Grawburg et al., 2014).

The ICF framework has also been used in a variety of ways within the autism field, including to develop goals (Rämä et al., 2019) and describe caregivers’ perceptions of their child's functional strengths and challenges (Viljoen et al., 2021). Researchers have also applied the ICF framework to describe the presentation of autism (De Schipper et al., 2016), further confirming its applicability to autism. The ICF framework has been recommended for identifying outcomes of caregiver-delivered programs because it captures children's functional behaviors, which have been identified as a priority for caregivers (McConachie et al., 2018).

#### Analysis

Interview transcripts were imported into NVivo Qualitative Data Analysis Software (2014), and an inductive/deductive hybrid codebook approach to thematic analysis was used to analyze the transcripts (Fereday & Muir-Cochrane, 2006). In this type of thematic analysis, a code book is developed based on an identified research question and a theoretical framework, in this case, the ICF framework, allowing for new themes and ideas to be uncovered in alignment with the guiding framework (Fereday & Muir-Cochrane, 2006). It has been suggested that taking a hybrid approach allows for the analysis process to be theory-driven, while also ensuring the voices of participants are heard (Proudfoot, 2023). Our multidisciplinary research team collaborated throughout the analysis process.

The analysis process included five steps. First, the first and fourth authors familiarized themselves with the data by reviewing all transcripts. Next, the research team developed an initial codebook using the ICF framework. After the codebook was developed, the first and fourth authors independently coded two transcripts and then met to review any inconsistencies and update the codebook accordingly. The fourth author then coded all the transcripts in NVivo using the updated codebook. The first author reviewed the coded transcripts throughout the coding process to ensure they followed the codebook. Preliminary themes and subthemes were developed and defined, and example quotes were selected to represent the perspectives of participants. These preliminary results were shared with the research team, and their feedback was incorporated to finalize the themes.

## Results

### Quantitative results

Quantitative results are presented in Table 2. Notable outcomes include 95% (*n *= 20) of participants feeling supported by their SLP and that program content was valuable “*to a fairly/extremely great extent*.” When asked if they thought their child's ability to communicate had improved, 57% (*n *= 12) said “*to a fairly/extremely great extent*,” but when asked if communication between the caregiver and the child had improved, most (*n *= 15, 71%) said to a “*to a fairly/extremely great extent*.” Almost all caregivers (*n *= 20, 95%) reported increased knowledge of communication strategies to use with their child. In terms of personal outcomes, 90% of caregivers (*n *= 19) said their confidence to teach their child communication skills had increased “*to a fairly/extremely great extent*.” All caregivers reported that the program helped them identify new opportunities for working on communication with their child.

**Table 2.** Caregivers’ responses to Likert scale items.

To what extent did you/would you… To what extend did the program…	Not at all (1) *N* (%)	To a very small/small extent (2–3) *N* (%)	To a moderate extent (4) *N* (%)	To a fairly/ extremely great extent (5–7) *N* (%)	Mode^a^ (Min, Max)
… find the information presented valuable?	0	0	1(5)	20 (95)	7 (4, 7)
… get the information you wanted?	0	0	2 (10)	19 (91)	7 (4, 7)
… use the communication strategies you learned with your child?	0	0	2 (10)	19 (91)	6 (4, 7)
… feel supported by the service providers (e.g., Speech Language Pathologists)?	0	0	1(5)	20 (95)	7 (4, 7)
…improve your child's ability to communicate?	0	3 (14)	6 (27)	12 (57)	5 (3, 7)
…improve communication between you and your child?	0	3 (14)	3 (14)	15 (71)	5 (3, 7)
…increase your knowledge about communication strategies to use with your child?	0	0	1(5)	20 (95)	5 (4, 7)
…help you to recognize other teaching opportunities you could use with your child?	0	0	0	21 (100)	5 (5, 7)
…increase your confidence around your ability to teach communication skills to your child?	0	0	2 (10)	19 (91)	5 (4, 7)
…change your approach to parenting?	0	3 (14)	2 (10)	16 (76)	5,6 (2, 7)
…feel satisfied with the overall Program?	0	0	1(5)	20 (95)	7 (4, 7)
…recommend this Program to a parent with a child who had similar communication challenges?	0	1 (5)	0	20 (95)	7 (3, 7)

*Note*. ^a^ If more than one mode, both listed.

### Qualitative results

**Figure 1.**
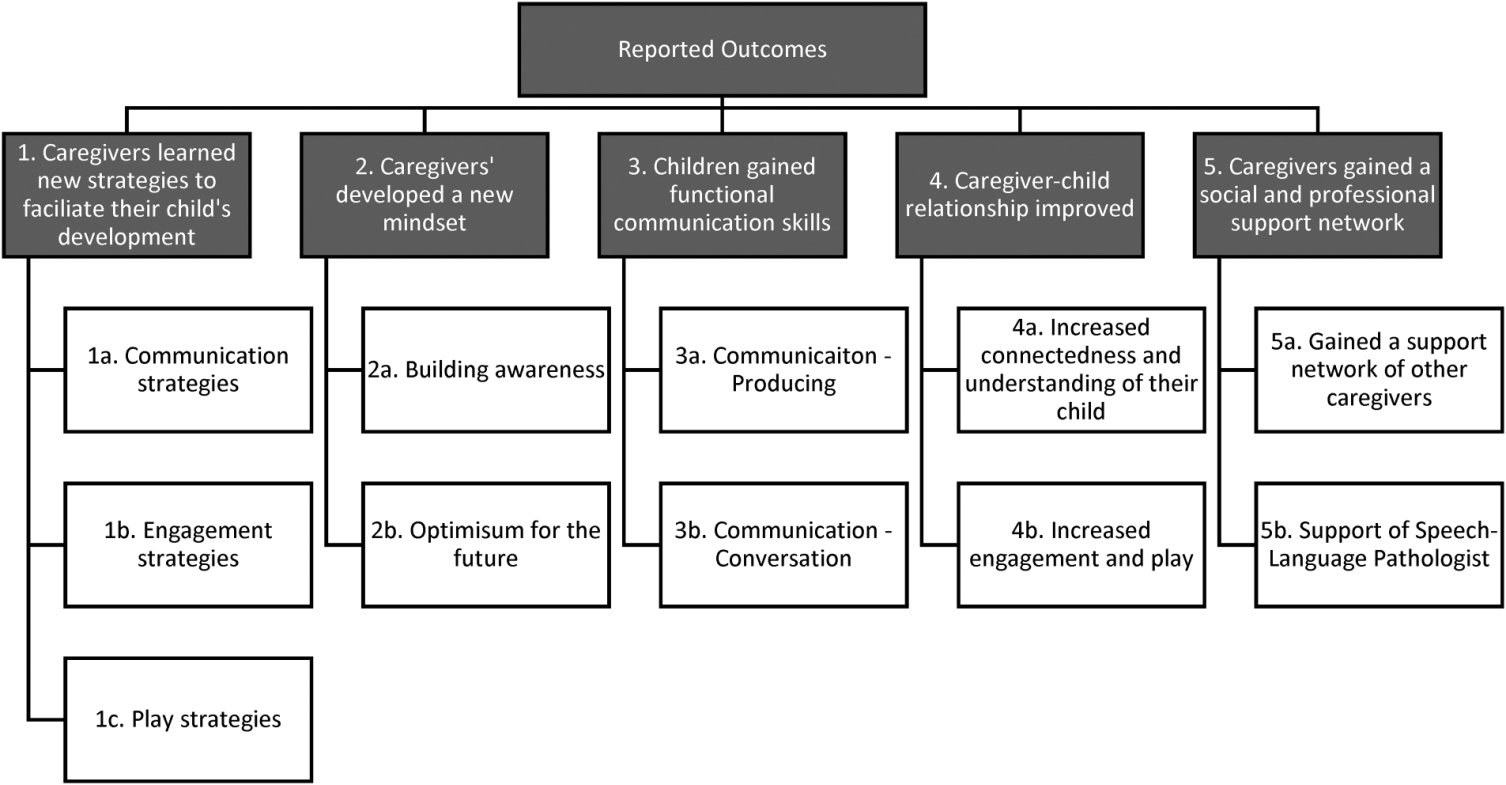
–—Caregivers’ reported outcomes following the virtual More Than Words^®^ program.

Five themes were identified during thematic analysis that represented caregivers’ perceived child and caregiver outcomes following the virtual MTW program: (1) caregivers learned new strategies to facilitate their child's development, (2) caregivers developed a new mindset, (3) children gained functional communication skills, (4) caregiver–child relationship improved, and (5) caregivers gained a social and professional support network (see Figure 1). These themes fell within four of the five parts of the ICF framework: Activities (Themes 1 and 3), Participation (Themes 3 and 4), Personal Factors (Theme 2), and Environmental Factors (Theme 5). No themes were identified within the Body Structures and Functions component. Themes and associated subthemes are described and illustrated with supporting quotes below.

#### Theme 1: Caregivers learned new strategies to facilitate their child's development

The first theme fits within the Activities section of the ICF framework and outlines the specific strategies caregivers reported learning during the virtual MTW program. Within this theme, three subthemes related to communication, engagement, and play strategies emerged.

##### Communication strategies

Caregivers reported learning two main strategies to support their child's communication development. The first was for caregivers to adjust their own use of language to provide their child with more opportunities to engage in interactions: “One of the strategies that really helped us was not asking so many questions that are yes and no questions. So, gearing questions more towards questions where my son actually has to give a response” (P104). Additionally, caregivers learned to ensure they were pausing while speaking and providing their child opportunities to engage in the conversation. The second strategy caregivers reported learning was the importance of positioning their bodies to ensure they were at their child's eye level. For some, this was not something they had previously considered as important for facilitating communication; however, through the program, they learned being face-to-face was a critical first step in setting up successful interactions.

[SLP] suggested how to communicate like where you should be sitting, eye contact and be at their level. Reading books, you know she would tell you how you should hold the book and what position you should be sitting in so that you can get the eye contact and get that communication going. (P105)

##### Engagement strategies

For this study, engagement strategies were defined as ways for caregivers to increase interactions with their child. Many caregivers reported that engagement with their child was a challenge prior to starting the program; however, through the program caregivers learned strategies to engage their child in social interactions. A commonly reported strategy was following the child's lead and joining their play, during which caregivers follow their child's interests rather than trying to direct the interaction: “playing alongside with him and making yourself kind of part of his world. We didn't know how to do that” (P102). The idea is that the caregiver is not directing the interaction, but instead joining in on something the child already enjoys, which can help promote engagement (Sussman et al., 2016). Some caregivers felt direct guidance from the SLP was helpful for learning to join in their child's play:

Before, my daughter is sitting by herself and playing by herself. Never any program explain to us we can join them. But then this program they said, we can go to sit next to them. (P118)

Learning to adjust their body positioning to increase their ability to engage with their child was also identified as an important strategy for facilitating engagement, such as being face-to-face with their child.

A lot of the time when I would be doing stuff with [child], I wouldn't be sitting face to face with him, I'd be more to the side. So [SLP] would tell me, why don't you try getting to his eye level and get face to face with him, you might get a better reaction that way, so that was, that was something that I always remember and stuck with me. (P107)

##### Play strategies

Caregivers learned to support their child's play skills by playing both with and without toys. For example, caregivers learned to set up the environment to support play development using strategies like limiting the toys available to avoid overwhelming their child: “I had too many toys in my living room, and it was overwhelming and distracting” (P101). Caregivers also reported learning to support play skill development without a physical toy by integrating games and songs into daily routines, which was a strategy that also supported communication development.

When I think of playing with my son, I think of playing with an object with a car or with a ball or paints or something, but it doesn't necessarily have to be that, and doing that is how he said his first word. I would chase him and I'd be like, I'll do a countdown 1-2-3 and then I'd wait, and then I'll say go and then he ended up saying ‘go’. (P111)

Another strategy was “playing alongside your child,” where caregivers learned to bring their own toy and model a new way to use it, instead of taking a toy from the child to demonstrate play. This strategy provided caregivers with the opportunity to teach their child new play skills without having to significantly disrupt the child's existing play routine.

One suggestion was that we have our own toys so we're not taking it from our child, we have our own set of blocks, or we have our own doll we're working with. So, we’re not taking their doll to show them how to use it or how to feed it while we're talking. We come with our own toys, we have our own book, and that was helpful. (P101)

#### Theme 2: Caregivers developed a new mindset

The second theme captures caregivers’ perceived outcomes that were not directly related to the program content but were instead viewed as personal gains from participating in the program. Two subthemes were identified: (a) building awareness and (b) optimism for the future, and both were considered to fall under the Personal Factors section of the ICF framework, which can include a variety of features related to an individual's background that can positively or negatively impact outcomes related to Activities and Participation. Both subthemes were seen as positively contributing to caregivers’ lives and their daily interactions with their child.

##### Building awareness

Caregivers reported becoming more aware of their behavior and how it could impact interactions with their child during the program. Individual video feedback sessions were identified as being especially helpful for caregivers to reflect on how they could adjust their use of communication facilitation strategies to further support their child. By reviewing video recordings with their SLP, caregivers gained insight into their behaviors. For example, many caregivers reported gaining awareness of how much they talked during interactions: “We didn't realize just how much we were talking, and so when we reviewed the video of the activity that we had done, it was like we weren’t giving [child] a chance to speak” (P102).

##### Optimism for the future

Caregivers also reported having new optimism for the future. Through learning the strategies, caregivers were excited and eager to support their child, and hopeful for the future: “Makes you happy, makes you hopeful that I know that this has a solution because when you apply those things that the [SLP's] telling you and see the child improve, there's hope” (P119). Learning about the stages of communication and watching videos of children at the different stages showed caregivers how children's communication skills could change and develop. Even if their child was not at the stage of the child in the video, caregivers reported feeling optimistic that they could help their child reach that next stage by implementing the strategies they learned in MTW: “Gives you that hope that like hey I can get my child there too” (P108). Finally, some caregivers noted that the overall aim of the program allowed them to focus on their child's unique strengths and created a positive outlook for the future: “It's all about bringing the best out of them” (P110).

#### Theme 3: Children gained functional communication skills

The third theme explores the program outcomes for the child. Within this theme, two subthemes were identified: (a) Communication—Producing and (b) Communication—Conversation. The ICF framework includes codes for communication in both the Activities and Participation sections. Communication as it relates to Activities is described as completing a task or action, whereas communication as it relates to Participation is more specific to engagement and includes involvement in life situations, including engagement in conversation (World Health Organization, 2001).

##### Communication—producing

Caregivers reported that their use of program strategies resulted in their child engaging in more verbal communication and producing more words: “We are stretching and repeating the words, it's very helpful. Now like she's repeating words. We learned this one from this program” (P118). Caregivers were excited about their child's new communication skills, even if gains were small: “now he's saying colors and, and actually stopping to think and saying words that weren't there before” (P109). Although some families reported an increase in verbal communication, others noted that gains in that area were not as noticeable as they had hoped.

##### Communication—conversation

In addition to increasing their expressive vocabulary, caregivers commented on gains in other communication skills that positively impacted children's involvement in social interactions. Reported social communication outcomes included increased social interactions, turn-taking, joint attention (including increased eye contact), and showing. As one caregiver described, “The back-and-forth communication has improved dramatically” (P104).

Before my daughter, she is just sitting down in the window and just looking, but now she understands when I sit with her, I say something. She then tried to communicate with us like she can make eye contact. (P118)

Another reported improvement was their child being better able to express needs and preferences. Through verbal communication or the use of gestures (e.g., pointing), caregivers were better able to understand their child's needs.

Now she can go to the pantry if she wants something to eat, put her hand on the fridge if she wants like something to drink, or she's even come to the point where she'll bring you like a water bottle if it's empty and be like, I want more or bring you the remote is her favorite. (P106)

Some caregivers also reported that their partner or extended family members had noticed improvements in their child's social communication skills: “The eye contact is a big deal. Even my husband he got teary eyed when she started, like, eye contact. My mother-in-law was like shocked. Everybody noticed a huge difference” (P106).

#### Theme 4: Improved caregiver–child relationship

The fourth theme captures program outcomes related to the caregiver–child relationship, and fits within the Participation component of the ICF framework, which includes a code for interpersonal interactions and relationships. While the MTW program aims to improve children's communication skills, caregivers reported that it also impacted the caregiver–child relationship. Within this theme, two subthemes were identified: (a) increased connectedness and understanding of their child and (b) increased engagement and play.

##### Increased connectedness and understanding of their child

Caregivers reported feeling more connected to and having a better understanding of their child, including new knowledge about their child's likes and dislikes. Through learning about the different forms of communication as well as strategies for how to better engage with their child, caregivers reported being more connected to their child.

More Than Words sort of let me go into her world and understand things from her point of view and be there with her and we have created a much deeper bond thanks to that. (P106)

As described in the first theme, caregivers learned strategies to improve their connection with their child, which positively impacted the caregiver-child relationship. For example, by learning how to adjust their communication style, caregivers experienced increased positive and joyful interactions with their child: “Once I started imitating her or would come to her when she's doing something like it's, it's quite remarkable how they light up and start to work back and forth with you that way” (P116). As children's communication skills improved, caregivers felt more connected because they better understood what their child needed.

##### Increased engagement and play

Caregivers also reported increased engagement with their child during daily routines as the strategies they learned could be easily implemented (e.g., during story time) and created more opportunities for positive engagement.

There was one instance where she had a book, and she wouldn't let me sort of get into it with her, so I sat in front of her with the same book, and I was flipping, and she just sort of looked to the side and smiled, and then she wanted to flip my book. (P106)

Caregivers also reported learning new ways of having enjoyable interactions that would also support their child's communication development. Example activities that were viewed as fun included games and songs.

[The child] loves songs and just learning that I can turn that into something else by stopping early in a line, so that she can fill in the blank and, you know, trying to get her to participate more and now she does it so much more. She has brought me a whole collection of books that are about songs and wanted to do that back and forth, so it was just really nice to see that she saw that it is okay, this is a way we can play together. (P116)

#### Theme 5. Caregivers gained a social and professional support network

The final theme encompasses two Environmental Factors that caregivers gained through participating in the virtual MTW program: (a) support from other caregivers and (b) support from the speech-language pathologist.

##### Support from other caregivers

Caregivers reported gaining a support network of other caregivers experiencing similar challenges who could relate to their situation. The MTW group sessions reportedly provided opportunities for caregivers to interact with one another, share stories, and hear how others were implementing the program strategies.

The benefits or advantages, to be in a program with other parents dealing with the same thing and like seeing or hearing different experiences and different methods that people were using. (P105)

Support from a peer group was an important outcome for families who may have been experiencing social isolation, which may have been particularly pronounced as they were participating during the COVID-19 pandemic.

I just found it like more of a community, because at first I was just feeling like is it only my child, and obviously I knew like other children are struggling with it, but when you're like just dealing with it by yourself, you just feel like, oh, it's only my child. (P115)

As a testament to the benefits of a peer group, many caregivers planned to stay connected with their new support network after the program ended through group chats and play dates.

##### Support from the SLP

Caregivers reported their SLP was available to listen to their concerns and support them as they applied new knowledge. SLPs were reported to provide encouragement and empower caregivers to feel like they could support their child's development.

[The SLP] was just super encouraging to us as parents, like she's spending an hour every couple weeks with our child, but we're the ones who are going to work with them, she really empowered us to do it and made us feel like we were equipped, we were the best people to do the job. (P101)

Although the MTW program includes group components, caregivers still felt that their SLP provided initial feedback specific to the needs and abilities of their child. Caregivers felt that their specific concerns were heard and addressed: “There could be one solution for one child in that problem and for my son it wouldn’t work. So, then she’ll find a way to help my son” (P111).

## Discussion

The purpose of this study was to understand caregivers’ perceived outcomes of the virtual MTW program in the context of the ICF framework. The outcomes identified were associated with multiple components of the ICF framework to capture the ways caregivers impacted their child by implementing the strategies taught. Outcomes identified include those directly associated with the content and purpose of the program (i.e., expected) and those indirectly related to program content (i.e., unexpected).

### Identified outcomes and the ICF framework

None of the five identified themes were associated with the Body Structures and Functions component of the ICF framework. Caregivers instead focused on their children's functional communication skills and interactions in daily activities, a perspective that aligns with the recommended shift away from the traditional biomedical approach of measuring outcomes related to children's impairments (World Health Organization, 2001). While clinicians are encouraged to use the ICF framework to target functional outcomes that are valued by caregivers (McNeilly, 2018), the literature suggests SLPs may not be adopting that model in practice. A recent scoping review of intervention goals reported in the literature suggested most goals for preschoolers with language difficulties and disorders were categorized in the Activities component of the ICF framework and fewer goals specific to Participation were identified (Kwok et al., 2022). While SLPs focus on outcomes specific to children's impairments, caregivers report prioritizing those related to Participation (Roulstone et al., 2013; Thomas-Stonell et al., 2009). This discrepancy between SLPs’ practice and caregivers’ preferences suggests there is a mismatch between how different groups view and prioritize child outcomes.

All caregiver-reported child outcomes in the current study fit within the Activities and Participation components of the ICF framework, suggesting caregivers were primarily concerned with how the program impacted their child's ability to use communication to interact with others in their daily lives. Previous research has reported that children gain specific social communication skills following the MTW program (Garnett et al., 2022a; Lok et al., 2021; Sokmum et al., 2017), but has not assessed whether or how those gains impact children's daily participation. MTW is described as a program that will support caregivers in achieving specific goals with their child, including increasing interactions, developing play skills, and increasing the child's understanding (The Hanen Centre, 2023). This description may be part of the explanation as to why functional outcomes most important to families are not included in the literature. Additionally, it may be due, in part, to the limited availability of suitable outcome measures. One outcome tool that may be appropriate for use with autistic preschoolers is the Focus on the Outcomes of Communication Under Six (FOCUS; Thomas-Stonell et al., 2013). The FOCUS is a caregiver-report outcome measure that can be used to assess changes in a child's communicative participation during speech and language interventions, and includes items that describe how children use their communication skills to engage in conversations and social interactions. Many previous studies on the MTW program have assessed vocabulary growth associated with program participation using the MacArthur-Bates Communicative Development Inventory (e.g., Girolametto et al., 2007; Prelock et al., 2011; Sokmum et al., 2017), but interestingly, very few caregivers in the current study commented on their child's expressive vocabulary outcomes. Caregivers in this study focused more on children's functional communication skills, including their non-verbal communication skills. As such, a tool like the Early Social-Communication Scales (Mundy et al., 2003) may be more useful than vocabulary measures for capturing the functional expressive communication skills identified as important by caregivers.

A primary aim of caregiver-delivered programs is to provide families with strategies they can implement in daily routines to support their child's development (Bearss et al., 2015), which caregivers in the current study felt they had learned. Within the Activities component of the ICF framework, caregivers’ use of new language facilitation strategies was commonly reported. As part of the MTW program, caregivers learn about the different forms communication can take, including those that are nonverbal, and thus caregivers are encouraged to respond to their child's communication attempts. To capture this program outcome, previous research specific to MTW has reported caregivers became more responsive and less directive when communicating and interacting with their child following the program (Carter et al., 2011; Garnett et al., 2022a; Girolametto et al., 2007; Sokmum et al., 2017). Interestingly, caregivers in the current study did not comment on gains specific to responsiveness, instead focusing on the strategies they learned and the new ways in which they could understand their child's communication. It is possible that responsiveness may not be the appropriate categorization, but rather how caregivers respond to their child communication, whatever form that takes. Using the term responsive could suggest that, initially, caregivers are not responsive to their child, when in fact they are just not aware of the various forms communication can take. Future research could consider assessing these important caregiver-identified outcomes alongside those related to responsiveness, but perhaps consider using another term that does not create a negative perception of caregivers.

Caregivers also noted gains related to Personal Factors within the ICF framework, including developing a new mindset. For example, the video-feedback sessions that are included as part of the MTW program were reported to provide caregivers with an opportunity to reflect on their use of communication facilitation strategies. While much of the available research on MTW has focused on caregivers learning new strategies, this study identified the importance of reflexivity as part of the learning process. The use of video-feedback sessions as an intervention model has been assessed in the literature and reported positive outcomes include increased participation from families during the intervention (Aiello et al., 2022) and increased caregiver self-efficacy (Poslawsky et al., 2015). However, further research is needed to understand the role of reflexivity in longer-term implementation success. Caregivers also reported optimism for the future as a program outcome because they saw the impact that strategy use could have on their child's communication skills.

In line with the available literature, caregivers reported gaining a new peer support network as a positive outcome within the Environmental Factors component of the ICF framework. Support from their SLP was a second identified outcome within the same component, a finding that is also aligned with previous research related to the MTW program (Denusik et al., 2023; Garnett et al., 2022b). To our knowledge, there are currently no validated tools that could measure these types of Environmental outcomes associated with speech-language pathology interventions. The development of a field-specific quantitative measure may be an important future research direction, given that group and professional supports have been identified as important in multiple studies on MTW (Denusik et al., 2023; Garnett et al., 2022b; Patterson & Smith, 2011) as well as more generally in research on providing child- and family-centered care (Henderson et al., 2016).

### Clinical implications

For SLPs delivering MTW, these results shed new light on the many ways in which families of young autistic children may benefit from participating in a caregiver-delivered program. In addition to their efforts to target children's impairments associated with Body Structures and Functions and Activities, SLPs could consider structuring intervention programs and goals to better target the outcomes identified as important by caregivers. Specifically, focusing on functional child outcomes associated with Activities and Participation, and caregiver outcomes within the Personal Factors and Environmental Factors components of the ICF framework would align with what caregivers value. Caregivers in the current study identified a wide variety of outcomes associated with caregiver-delivered programs that should be considered when developing and implementing them. For example, gaining a support network was important to caregivers, thus clinicians should consider how they facilitate sessions to provide caregivers opportunities to connect with others. Similarly, developers could consider directly addressing personal outcomes for caregivers in addition to the traditional focus on the autistic child's communication development.

### Research implications

Caregivers were interviewed shortly after they had completed the virtual MTW program, so we were unable to comment on their longer-term perspectives on program outcomes. It would be important to understand the extent to which caregivers continue to implement the strategies they learn and the changes they observe in their child. This would provide critical evidence on whether and how caregiver-delivered programs continue to benefit families once the program has ended. A few studies have followed up with families approximately 5 months after their involvement in an MTW program to explore longer-term impacts and found that caregivers continued to implement strategies and children continued to gain new communication skills (Prelock et al., 2011; Sokmum et al., 2017); however, these studies had small sample sizes and were focused on researcher-selected outcomes. It would therefore be of interest to explore the longer-term outcomes associated with caregiver-delivered programs using qualitative methods to identify and prioritize new research directions for outcome assessment. Finally, while this study explored caregivers’ perceived outcomes, the SLPs delivering the program may have a different perspective on program outcomes for children and caregivers. It would be of interest to explore how SLPs’ reported outcomes align with the ICF framework components and whether and how they are similar to those reported by caregivers.

By taking a hybrid inductive/deductive analysis approach and using the ICF framework, we were able to showcase a broad range of outcomes associated with the virtual MTW program that caregivers viewed as important, but are not often assessed in autism intervention research. While these outcomes may not be the primary aims of caregiver-delivered programs like MTW, they were impactful enough that families shared them with us. The discrepancy between caregivers’ perspectives and researchers’ and clinicians’ focus on outcomes suggests it is critical to engage end users in the research process so their perspectives can be integrated to create programs and services that meet their needs. With many interventions available to families, especially under the caregiver-delivered umbrella, it is important to ensure services address families’ needs and preferred outcomes. Future research could focus on the development and validation of new tools to accurately capture outcomes within the more difficult-to-measure ICF components such as Participation, Personal Factors, and Environmental Factors. However, any such tools should be developed in collaboration with caregivers to ensure they are capturing the outcomes that are important for families. Similarly, new research could focus on the co-creation of an evidence-informed evaluation framework that could be used to assess outcomes that are important for caregivers. Such a framework could guide researchers developing and evaluating speech-language pathology programs for autistic preschoolers and their caregivers to ensure they are meeting the needs of their end users.

### Limitations

This study was a secondary analysis of data from interviews that were conducted to understand caregivers’ perceived barriers and facilitators to participating in the virtual MTW program during the COVID-19 pandemic. It is important to note that the interview questions did not explicitly ask about perceived program outcomes; however, the use of a semistructured interview format with open-ended questions allowed families to comment on a wide variety of topics and let us extract important information about their perceived program outcomes. Additionally, as caregivers participated in the program during the pandemic, it is possible participants’ experiences and perceived outcomes were different from those who participated in the virtual program under more typical circumstances. Most of our families were middle- and high-income earning and lived in an urban setting. While it has been suggested that virtual programs increase accessibility for those in rural communities, more research is needed to see if and how perceived outcomes of the program vary for different types of families. In particular, virtual programming assumes families have internet access and the technology required to best participate (e.g., desktop computer); however, that is not always the case. Understanding the varying needs of all families is critical to ensuring services meet families’ diverse needs.

Finally, it is possible that there was a social desirability bias, where caregivers only wanted to share positive outcomes (Bergen & Labonté, 2020). No caregivers identified negative or adverse effects of the program; however, they were not explicitly asked. With new research emerging about the adverse effects of some autism interventions (e.g., Applied Behavioral Analysis; Kupferstein, 2018; McGill & Robinson, 2021), future work should directly ask about negative outcomes to provide a complete picture of a program's impact.

## Conclusions

While research on the impact of MTW has been explored using a variety of quantitative outcome tools, our study explored caregiver-reported outcomes qualitatively. By engaging caregivers to share their perspectives on the virtual MTW program, new functional outcomes not previously reported in the literature were identified. Caregivers’ reported outcomes focused primarily on children's functional daily communication skills as well as important Personal and Environmental outcomes following program participation. Results will be important for researchers developing or evaluating caregiver-delivered programs for caregivers of autistic children. Clinically, this study provides new perspectives on important impacts of clinical services for autistic children and their families, and on intervention goals that may be most meaningful for caregivers.

## Appendix A: Interview Questions

*Warm-up*

As you know, in this research we’re interested in understanding the experiences of parents participating in the More Than Words (MTW) program during COVID-19. Before we get to that, I wonder if we could begin by having you tell me a bit about yourself and your family?

- Tell me a little bit about yourself and your family

1. Do you recall how you first heard about the MTW program?
  [Potential prompts]
  • For someone that does not know anything about the MTW program, how would you describe it?
  • How did you first get involved with the program?
  • Besides you and your child(ren) are other people in your family involved?
2. During COVID-19 the MTW program is being delivered virtually and not in person. I’m curious to know what this experience has been like for you and your family. Can you provide me with a description of your experience?
  [Potential prompts]
  • What have been some of the unique things that you have experienced while being part of the MTW program?
  • What were some of the benefits or advantages you feel that you or your family experienced being part of the virtual MTW program?
  • What are some of the not-so-positive things or disadvantages you feel that you or your family experienced being part of the virtual MTW program?
3. Did you experience any challenges with technology while participating in the virtual MTW program? (Technology includes videoconferencing, connectivity challenges, video camera)
  [Potential prompts] *If Yes*
  • Can you describe the challenges you faced at the beginning, when adjusting to the virtual program
  • How did the technological challenges change over time?
  • Was there ever an instance when a technology problem made it hard for you to participate in the program?
4. Here's a different sort of question – What three emotions do you feel when you think about how the speech-language pathologist (SLP) shared knowledge or information with you during your time in the MTW program? Can you tell me a bit more about why those emotions come to mind?
  [Potential prompts]
  • Can you describe a time when the SLP helped you?
  • What did the SLP do to make you feel supported?
  • How did they help you feel competent? Did you ever feel incompetent? Tell me more about that emotion.
  • Can you describe a time when a miscommunication occurred?
5. What did the SLP do to make you feel competent during your virtual MTW appointments? Was there something they ever said or that happened that made you feel incompetent?
6. In what ways did the SLP provide you with opportunities to provide input on the activities you were asked to do or the activities you planned to do at home with your child as part of the MTW program?
  [Potential prompts]
  • What was your experience receiving feedback from the SLP about the activities you planned or did with your child in this virtual environment?
  • What kinds of suggestions did the SLP provide to try to help you during your time together in this virtual program?
  • How did the SLP guide you in implementing the MTW program in your home?
7. I would like to hear in your own words the ways in which the SLP supported your child's emerging language and communication skills during your time in the program.
  [Potential prompts]
  • Can you tell me some of the things that you remember that the SLP suggested you try and why you think they made those suggestions?
  • What kinds of things, if any, did they do to support you?
  • What kinds of tools/techniques did they use to support you?
8. We’re interested in hearing from you about your relationship with the SLP you met through the MTW program.
  [Potential prompts]
  • Do you remember the types of things the SLP did, or said to you, that made you feel that you were cared for even while receiving the MTW program virtually? If you don’t feel that you were in a caring atmosphere, what do you think the SLP did or say to make you feel that way?
9. In thinking about your time in the MTW program is there anything the SLP could have done (or could do) to make your time in the program better for your child or your family?
10. Your SLP spent some time observing you implementing strategies from the MTW program with your child. What was your experience like being observed by the SLP in a virtual fashion?
11. Here's a follow-up question: How do you think this might have been different if you were meeting in person/face-to-face?
12. At each MTW session, your SLP provided you with some kind of feedback. What was it like receiving feedback from the SLP? Can you describe your experience of receiving feedback in this virtual environment?
13. How do you think this might have been different if you were meeting in person/face-to-face?
14. During the MTW program you had the opportunity to interact with other parents in the program. What was that experienced like?
  [Potential prompts]
  • Were there things you learned from other parents that were different from what the SLP taught?
  • How do you feel this connection would have differed in person?
  • Were there benefits (drawbacks) to having other parents in the group?
15. List the ways in which you were satisfied with the MTW program, also telling me what happened to make you satisfied.
  [Potential prompts]
  • List the ways in which you were satisfied with the virtual delivery of the MTW program, also telling me what happened to make you satisfied.
  • Similarly, List the ways in which you were not satisfied with the virtual MTW program, also telling me what happened to make you dissatisfied.
16. If a parent asked you to relate to them your experiences in the MTW program, what would you say?
  [Potential prompts]
  • If a parent was starting the program tomorrow, what advice would you share with them?
17. What, if anything, has surprised you the most about participating in the MTW program virtually rather than face-to-face?
18. Let's imagine you were asked to advocate for keeping the MTW program virtual. What would be the top 2 reasons you would give for why the program should be virtual and why?
19. Let's imagine you were asked to advocate for having families receive the MTW program face-to-face. What would be the top 2 reasons you would give for why the program should be delivered face-to-face and why?

*Wrap-up*

Thank you so much for sharing your insights and experiences with me. Is there anything more about your experience in the MTW program in general or in participating in the MTW program virtually that we haven’t talked about yet that you would like to share or that you feel is important for us to know about in this research?
